# High Glucose Contribution to the TCA Cycle Is a Feature of Aggressive Non–Small Cell Lung Cancer in Patients

**DOI:** 10.1158/2159-8290.CD-23-1319

**Published:** 2025-02-17

**Authors:** Ling Cai, Nia G. Hammond, Alpaslan Tasdogan, Massar Alsamraae, Chendong Yang, Robert B. Cameron, Peiran Quan, Ashley Solmonson, Wen Gu, Panayotis Pachnis, Mayher Kaur, Brianna K. Chang, Qin Zhou, Christopher T. Hensley, Quyen N. Do, Luiza Martins Nascentes Melo, Jessalyn M. Ubellacker, Akash Kaushik, Maia G. Clare, Isabel N. Alcazar, Katarzyna Kurylowicz, Joseph D. Marcuccilli, Gabriele Allies, Andrea Kutritz, Joachim Klode, Vijayashree Ramesh, Thomas J. Rogers, Aparna D. Rao, Hannah E. Crentsil, Hong Li, Fang Brister, Phyllis McDaniel, Xiaohong Xu, Bret M. Evers, Lauren G. Zacharias, Jessica Sudderth, Jian Xu, Thomas P. Mathews, Dwight Oliver, John D. Minna, John Waters, Sean J. Morrison, Kemp H. Kernstine, Brandon Faubert, Ralph J. DeBerardinis

**Affiliations:** 1 Children’s Medical Center Research Institute, University of Texas Southwestern Medical Center, Dallas, Texas.; 2 Quantitative Biomedical Research Center, Peter O’Donnell Jr. School of Public Health, University of Texas Southwestern Medical Center, Dallas, Texas.; 3 Section of Hematology and Oncology, Department of Medicine, University of Chicago, Chicago, Illinois.; 4 Department of Dermatology, University Hospital Essen & German Cancer Consortium, Essen, Germany.; 5 Department of Radiology, University of Texas Southwestern Medical Center, Dallas, TX, USA.; 6 Clinical Research Unit, University of Texas Southwestern Medical Center, Dallas, Texas.; 7 Department of Pathology, University of Texas Southwestern Medical Center, Dallas, Texas.; 8 Howard Hughes Medical Institute, University of Texas Southwestern Medical Center, Dallas, Texas.; 9 Hamon Center for Therapeutic Oncology Research, Department of Internal Medicine, University of Texas Southwestern Medical Center, Dallas, Texas.; 10 Department of Pharmacology, University of Texas Southwestern Medical Center, Dallas, Texas.; 11 Department of Cardiovascular and Thoracic Surgery, University of Texas Southwestern Medical Center, Dallas, Texas.; 12 Department of Pediatrics, University of Texas Southwestern Medical Center, Dallas, Texas.; 13 Eugene McDermott Center for Human Growth and Development, University of Texas Southwestern Medical Center, Dallas, Texas.

## Abstract

In patients with non–small cell lung cancer (NSCLC), the relationship between tumor metabolism and clinical outcomes is unknown. Here, ^13^C-labeled nutrients were intraoperatively infused into more than 90 patients with surgically resectable pulmonary lesions, and metabolic properties of resected tumors were correlated with survival. In NSCLCs infused with ^13^C-glucose, high ^13^C enrichment in tricarboxylic acid (TCA) cycle intermediates conferred a HR of 3.8 for early death, typically with metastasis. To test whether these features reflect requirements for metastasis, we generated patient-derived xenografts that spontaneously metastasize to multiple organs. Treatment with an electron transport chain (ETC) inhibitor reduced glucose-derived TCA cycle labeling but did not suppress subcutaneous tumor growth. However, ETC blockade reduced the abundance of circulating cancer cells and suppressed xenograft metastatic burden in distant organs. Our data demonstrate that isotope labeling can identify metabolic properties associated with metastasis in patients and that blocking the ETC suppresses metastasis in mice.

**Significance:**

Intraoperative ^13^C-glucose infusions in patients with NSCLC show that tumors with high labeling of TCA cycle intermediates progress rapidly, resulting in metastasis and early death. Blocking this pathway suppresses metastasis of human NSCLC cells in mice.

## Teaser

A longitudinal clinical study using intraoperative ^13^C-glucose infusions in patients with non–small cell lung cancer finds that use of glucose by tumors to supply the tricarboxylic acid cycle predicts poor overall survival.

## Introduction

Metabolic reprogramming is a hallmark of malignant cell growth (1). Metabolic plasticity—the ability to alter metabolism based on microenvironmental constraints—allows tumor cells to survive adverse conditions such as nutrient limitation, oxidative stress, and hypoxia (2). These adaptations are important during metastasis, in which tumor cells undergo a multistep process to escape the primary environment, travel through blood or lymph, colonize distant organs, and grow at these remote sites (3, 4). Elucidating tumor cell–intrinsic metabolic adaptations that support metastasis could uncover liabilities to suppress cancer progression.

Mitochondria are central hubs for generating energy and biosynthetic intermediates (5). In cancer cells, the importance of mitochondrial metabolism has often been overlooked in favor of increased glycolysis (the Warburg effect). High glycolytic rates in most cancer cells produced the misconception that mitochondria and oxidative phosphorylation (OXPHOS) are damaged in cancer or counterproductive for tumor growth (6). This concept has been challenged by studies *in vivo*, in which tumors oxidize multiple fuels in the mitochondria (7, 8) and ablation of components of the electron transport chain (ETC) suppresses xenograft growth (9, 10). Other preclinical studies indicate a selection for OXPHOS as tumors progress, including progression to metastatic cancer (11–14).

Tumor metabolism can be studied directly in patients by infusing stable isotope–labeled nutrients (e.g., [^13^C]glucose), before or during surgical resection of the tumor. To date, all tumor types examined using this method demonstrate oxidation of glucose-derived pyruvate in the tricarboxylic acid (TCA) cycle (15–18). Other circulating fuels, including acetate (19), glutamine (20), and lactate (21), can also be oxidized in at least some tumors. These metabolic features are heterogeneous across patients and tumor types (22, 23), raising the possibility that metabolic activity, as reported by ^13^C infusion, may correlate with heterogeneous clinical parameters. Whether tumor metabolism relates to outcomes, particularly patient survival, remains unclear. To answer these questions, we need to study a sizable group of patients who have undergone ^13^C infusions and track them over an extended period.

Over a span of 11 years, we administered ^13^C-labeled nutrients in more than 90 patients with pulmonary lesions, during surgical resection of these lesions. Metabolic properties of the tumors were then compared with clinical features, including outcomes. Our findings indicate that increased ^13^C enrichment in the TCA cycle is associated with a poorer prognosis in patients with non–small cell lung cancer (NSCLC). Additionally, we demonstrate that patient-derived xenografts (PDXs) generated from these tumors maintain levels of TCA cycle labeling similar to the levels observed in NSCLCs from patients, spontaneously metastasize to distant organs, and that both TCA cycle labeling and metastasis are blocked by ETC inhibition.

## Results

### ^13^C Enrichment in the TCA Cycle Distinguishes Tumors from Adjacent Lung

**Figure 1.**
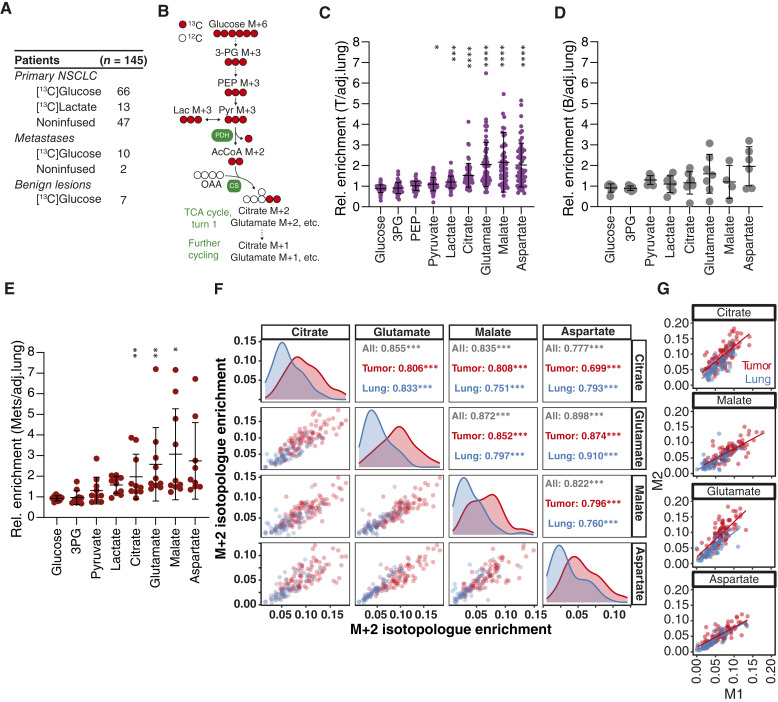
^13^C enrichment in the TCA cycle distinguishes tumors but not benign pulmonary lesions from the adjacent lung. **A,** Summary of patients with pulmonary lesions. **B,** Schematic of labeling from [U-^13^C]glucose, including glycolysis and multiple turns of the TCA cycle. **C–E,**^13^C enrichment comparisons between each lesion type and the adjacent lung. The sum of isotopologues of each metabolite is normalized to the corresponding metabolite enrichment in the adjacent lung. Each dot represents one patient. (**C**) Primary NSCLC tissues (*n* = 64; two additional patients with NSCLC provided tumor but not adjacent lung tissue and are not included in this analysis), (**D**) benign lesions (*n* = 7), and (**E**) metastatic tumors (*n* = 10). **F,** Spearman correlation analysis of ^13^C enrichment values between TCA cycle metabolites in each tissue type. Values from each sample (blue: lung; red: NSCLC tumor) are plotted to compare labeling between each pair of metabolites. **G,** Correlations between M+1 and M+2 isotopologues in NSCLC tumors (red) and adjacent lungs (blue). Data are mean ± SD. Statistical significance was assessed using *t* tests to compare tissue types. Significance in **C**–**E** was calculated with the Wilcoxon signed-rank test. Multiple comparisons were adjusted using the Holm-Sidak method. 3PG, 3-phosphoglycerate; AcCoA, acetyl-CoA; CS, citrate synthase; Lac, lactate; OAA, oxaloacetate; PDH, pyruvate dehydrogenase; PEP, phosphoenolpyruvate; Pyr, pyruvate. *,P<0.05; **,P<0.01; ***, P<0.001; ****, P<0.0001.

Patients with lung lesions suspicious of NSCLC were recruited for our study (NCT02095808). These lesions were analyzed through preclinical imaging, intraoperative ^13^C nutrient tracing, and postoperative analysis of metabolic and molecular features. Supplementary Table S1 contains clinical information from all recruited patients, including those from previous studies (21, 22), in which data are included in the analyses presented here. We expanded the cohort to include [U-^13^C]glucose infusions in patients with primary, treatment-naïve NSCLC (*n* = 66) and other pulmonary lesions (see below). Additional patients were infused with ^13^C-lactate (*n* = 13), and others provided tissue but were not infused (*n* = 47; Fig. 1A). In patients with primary NSCLC, metabolites extracted from the tumor and adjacent lung displayed comparable ^13^C enrichment in glycolytic intermediates relative to the total glucose enrichment (Fig. 1B and C). However, NSCLC samples displayed higher total ^13^C enrichment in pyruvate, lactate, and TCA cycle metabolites than the adjacent lung (Fig. 1C), in agreement with previous findings (21, 22).

Not all patients had NSCLC (Fig. 1A; Supplementary Fig. S1A). Histological analysis after surgery revealed that seven ^13^C-infused patients had benign lesions. Unlike NSCLC, labeling in benign lesions was indistinguishable from the adjacent lung (Fig. 1D). Several other patients (*n* = 10 infused with ^13^C) had pulmonary metastases from diverse primary tumors (Supplementary Fig. S1A). These metastases displayed elevated ^13^C enrichment in TCA cycle metabolites (Fig. 1E). Unlike the NSCLCs, TCA cycle intermediate labeling was as high or higher in every metastatic lesion compared with the adjacent lung. All patient isotopologue data are provided in Supplementary Table S2.

Labeling in metabolites extracted from tissues may reflect the metabolism of [U-^13^C]glucose within the tissue or metabolism of [U-^13^C]glucose elsewhere in the body, secretion of labeled metabolites into the bloodstream, and import of these secondary tracers by the tumor (24). Local metabolism should produce consistent labeling of different metabolites from the same pathway. We applied this logic to the TCA cycle by performing pairwise correlational analysis of M+2 labeling in citrate, malate, glutamate, and aspartate from each lung or tumor fragment from [U-^13^C]glucose-infused patients. M+2 labeling in these intermediates primarily results from [U-^13^C]pyruvate being decarboxylated by pyruvate dehydrogenase and the resulting [1,2-^13^C]acetyl-CoA entering the TCA cycle (Fig. 1B). This analysis revealed strong correlations among all metabolites in both lung and tumor fragments, consistent with local oxidation of glucose-derived pyruvate (Fig. 1F). M+1 labeling also correlated among these metabolites (Supplementary Fig. S1B); this pattern correlates with M+2 labeling and likely arises from multiple turns of the TCA cycle (Fig. 1B and G; ref. 25). Compared with lung, the tumors also contained higher M+3 labeling in TCA cycle intermediates normalized to M+3 pyruvate, consistent with the reported activation of pyruvate carboxylase in human NSCLC (Supplementary Fig. S1C; ref. 26). Elevated labeling of these metabolites was not a consequence of lower metabolite abundance because labeling and abundance tended to correlate positively in the tumors (Supplementary Fig. S1D).

**Figure 2.**
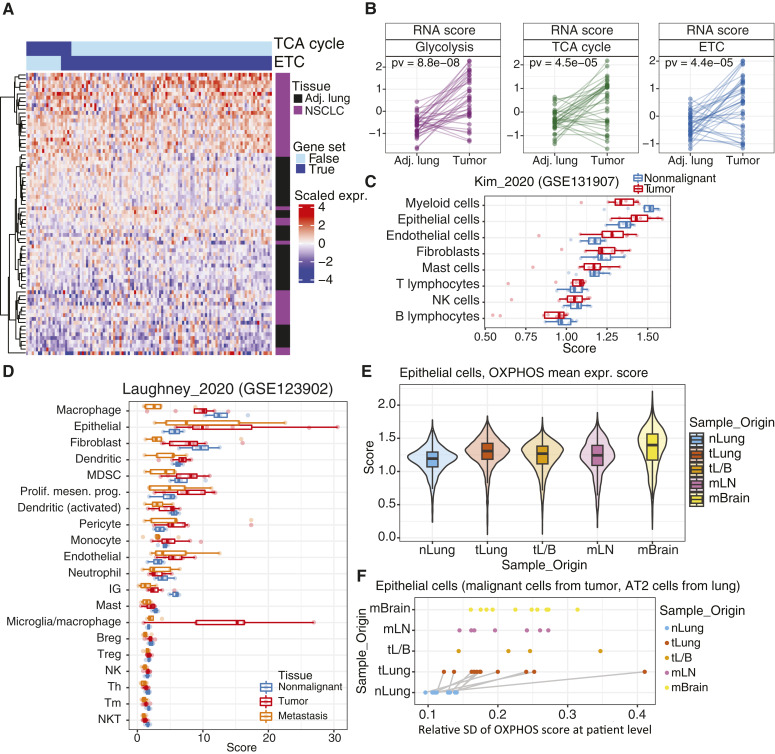
Cancer cells drive an OXPHOS expression signature in tumors. **A,** Heatmap of TCA cycle and ETC transcript differences between primary NSCLC and adjacent lung samples. **B,** RNA scores comparing matched tumor and adjacent lung for the pathways indicated. **C,** Single-cell RNA-seq data from GSE131907. OXPHOS scores were calculated as the mean expression of OXPHOS genes for each cell type, derived from tumor samples (red) or adjacent lung samples (blue). **D,** Single-cell RNA-seq data from GSE123902. OXPHOS scores were calculated as in **C** for each cell type, derived from nonmalignant lung tissue (blue), primary NSCLC tumors (red), or metastatic lesions (orange). **E,** Distribution of OXPHOS expression scores among epithelial cells from each sample type. **F,** Relative SDs of OXPHOS scores among epithelial cells from each sample type. Patient-matched normal lung (nLung) and NSCLC tumors (tLung) are connected by lines. Other sample types in **E** and **F** are tumors from late-stage biopsy (tL/B), metastatic lymph node (mLN), and brain metastases (mBrain). Breg, Regulatory B cell; MDSC, myeloid-derived suppressor cell; Prolif. mesen. prog., proliferating mesenchymal progenitor cell; Treg, regulatory T cell; Th, helper T cell; Tm, memory T cell; NK, natural killer cell; NKT, natural killer T cell.

Consistent with the higher TCA cycle labeling, RNA sequencing (RNA-seq) revealed higher expression of genes related to OXPHOS (specifically, the TCA cycle and ETC, Fig. 2A and B; Supplementary Table S3). Genes related to glycolysis also display enhanced expression (Fig. 2B). To assess the relative expression of OXPHOS-related genes among different cell types, we examined published single-cell RNA-seq data from human NSCLC. This revealed OXPHOS gene expression in many cell types in both the nonmalignant lung and the tumor. In the nonmalignant lung, the OXPHOS expression score was highest in myeloid cells, with a somewhat lower score in epithelial cells (Fig. 2C and D). In the tumors, however, this relationship was reversed, with epithelial cells (i.e., lung cancer cells) expressing the highest score.

OXPHOS and TCA cycle genes are heterogeneously expressed among individual cells. In epithelial cells, the OXPHOS score is much more heterogeneous among cells from tumors compared with cells from the lung (Fig. 2E and F). We performed additional analyses to assess the abundance and OXPHOS scores among specific myeloid and epithelial cell subtypes (Supplementary Fig. S2A and S2B; ref. 27). Within epithelial subtypes, tS2 and tS3 displayed the highest median OXPHOS scores. For myeloid subtypes, alveolar macrophages had the highest OXPHOS expression although their abundance was much lower in the tumor than the lung. To better estimate macrophage abundance, we conducted IHC staining to generate H-scores for the macrophage marker CD68, and we explored the correlation between metabolic measurements and macrophage abundance. Although many macrophage identifiers (e.g., CD68 H-score and transcriptome-inferred macrophage scores) correlated positively with each other, none correlated with TCA cycle labeling (Supplementary Fig. S2C). We also quantified CD68^+^ cells on our IHC images to estimate the contribution of these cells to the TME (Supplementary Fig. S2D and S2E). The macrophage content estimated from this method closely correlated with H-scores determined by a pathologist (Supplementary Fig. S2F), with a median macrophage content of 6%. Representative tumor images with varying levels of CD68^+^ cells are shown in Supplementary Fig. S2G.

Altogether, these data are consistent with extensive TCA cycle metabolism in the tumors, with malignant cells contributing to the labeling of TCA cycle intermediates. This analysis does not rule out the possibility that some label arises from the import of ^13^C-lactate generated from ^13^C-glucose (21). It also does not rule out the labeling contributions from nonmalignant cell types. However, in these samples, the relatively low abundance of myeloid cells and lack of correlation between myeloid gene expression and TCA cycle labeling suggest that myeloid cells are not the driver for the observed TCA cycle labeling.

### ^13^C Enrichment in the TCA Cycle Correlates with Poor Survival

**Figure 3.**
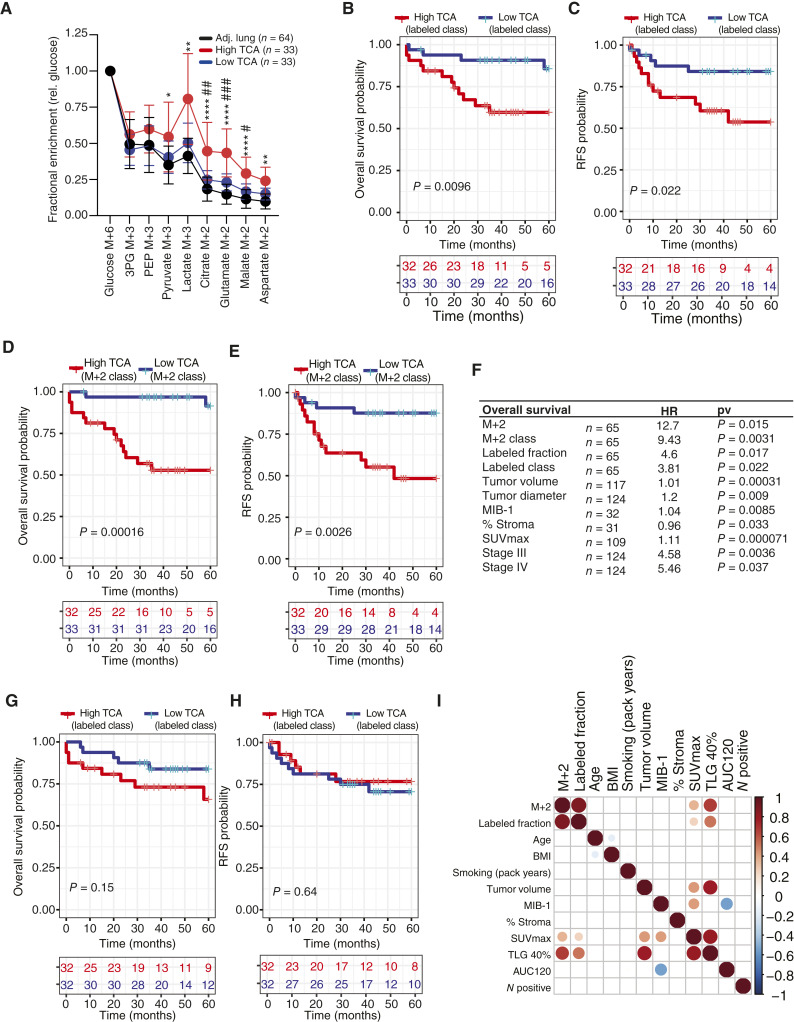
Increased ^13^C enrichment in the TCA cycle predicts reduced survival. **A,**^13^C enrichment in the adjacent lung and tumors with high or low TCA cycle labeling. Fractional enrichments of glycolytic (M+3) and TCA cycle (M+2) metabolites are normalized to enrichment of glucose (M+6) within the tissue. **B** and **C,** Overall (**B**) and recurrence-free (**C**) survival in patients whose tumors have high or low TCA cycle labeling. The groups include tumors above or below the median for total ^13^C enrichment in citrate, glutamate, and malate. **D** and **E,** Overall (**D**) and recurrence-free (**E**) survival in patients whose tumors have high or low M+2 enrichment. **F,**^13^C labeling and clinical factors and relationship to overall survival. **G** and **H,** TCA cycle labeling fractions in the adjacent lung (calculated and grouped as in **B** and **C**) do not correlate with overall (**G**) or recurrence-free (**H**) survival. **I,** Spearman correlation analysis of labeling features (M+2 and labeled fraction) with clinical metrics. Differences in ^13^C enrichment (**A**) were determined using a Kruskal–Wallis test with Dunn’s test. Survival differences were assessed by the log-rank (Mantel–Cox) test. BMI, body mass index; MIB-1, Mindbomb Homolog-1; SUV_max_, maximum FDG-PET standardized uptake value; TLG 40%, total lesion glycolysis, 40% threshold; AUC120, total area under the curve at 120 seconds, calculated from dynamic contrast-enhanced MRI; N positive, lymph node positive. *,P<0.05; **,P<0.01; ****, P<0.0001. (High TCA vs Adj. Lung), #, P<0.05; ##, P<0.01.; ###, P<0.001) (Low TCA vs. Adj. Lung)

The 11-year study allowed us to ask whether metabolic features in primary NSCLCs correlate with clinical outcomes. The consistently elevated TCA cycle labeling in lung metastases prompted us to investigate if this feature correlates with primary NSCLC aggressiveness. We assessed M+2 labeling of TCA cycle intermediates relative to glucose M+6 enrichment in the same sample. We also created an overall enrichment metric by summing the total labeling of TCA cycle–related intermediates [i.e., (1-unlabeled) for citrate, glutamate, and malate] and normalized this value to glucose enrichment. The tumors were then dichotomized into “high” and “low” labeling groups based on whether this enrichment metric was above or below the median for the cohort (Fig. 3A; Supplementary Fig. S3A and S3B). Using the summed TCA cycle enrichment values, both overall (Fig. 3B) and recurrence-free (Fig. 3C) survival were markedly worse among patients whose tumors had high TCA cycle labeling. The same was true when outcomes were followed according to M+2 labeling (Fig. 3D and E). Recurrence was defined as the appearance of a tumor in the remaining lobes of the lung or extrathoracic metastasis. Most patients who died had recurrent NSCLC (*n* = 25), usually through distant metastasis, whereas a few (*n* = 6) died with unknown status of recurrence. Under univariate analysis, the HR from high TCA cycle enrichment for overall survival was between 3.8 and 12.7, depending on the labeling metric (Fig. 3F; see the complete list of HRs in Supplementary Table S4). Although the patients also demonstrated heterogeneous labeling in the adjacent, nonmalignant lung (Fig. 1F), TCA cycle labeling in the lung did not correlate with outcomes (Fig. 3G and H).

We observed no association between tumor TCA cycle labeling and body mass index, age, sex, histological subtype, driver mutation, grade, or smoking history (Fig. 3I; Supplementary Fig. S3C–S3J). Similarly, TCA cycle enrichments in the adjacent lung tissue were not related to these clinical features (Supplementary Fig. S3K–S3M). Labeling did not differ among the lung samples when they were dichotomized by the metabolic class of the tumor (Supplementary Fig. S3N). TCA cycle labeling in the tumors correlated with markers of [^18^F]fluoro-2-deoxy-D-glucose (FDG) uptake on FDG-PET (Fig. 3I; Supplementary Fig. S3O and S3P) and maximum FDG-PET standardized uptake value correlated with poor overall survival (Fig. 3F). In a metabolomics analysis of more than 600 metabolites and after controlling for multiple comparisons, none of the metabolomics features, including TCA intermediates, were associated with overall survival (Supplementary Fig. S4).

### Generation of NSCLC PDXs Capable of Spontaneous Metastasis

Because most patients who experienced cancer progression had metastatic disease, we sought to explore a causal connection between metastasis and TCA cycle labeling. We established new PDX models derived from patients in this cohort, including patients infused intraoperatively with ^13^C. A complication of transplanting human tissue into immunocompromised mice is the unintended generation of human B-cell lymphomas (28, 29). To address this, we utilized histology and flow cytometry with antibodies against human CD45 (hCD45, a human pan-hematopoietic marker) to monitor contamination of B-cell lymphoma (Supplementary Fig. S5A and S5B). Any contaminated PDXs were purified by flow cytometry–assisted cell sorting (FACS) of HLA-positive, hCD45-negative cells before reinjection (Supplementary Fig. S5B, right). Once the PDXs were verified to be solely NSCLC, they were analyzed further. In some cases, PDXs were transduced with a dsRED–luciferase construct. Using this approach, we generated 10 PDXs from 62 tumor fragments derived from 52 patients, including four PDXs from patients with NSCLC and six from patients with lung metastases.

**Figure 4.**
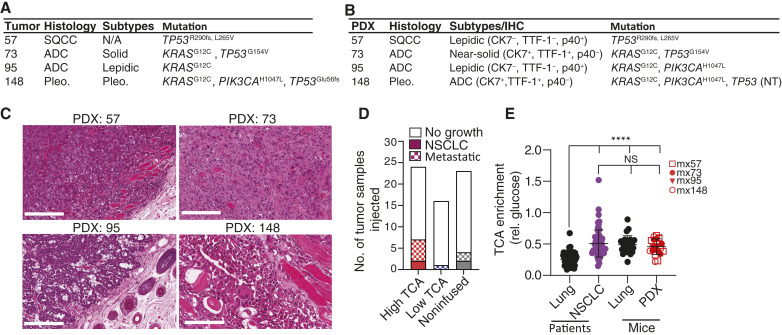
PDXs derived from primary NSCLC retain histological, molecular, and metabolic characteristics. **A** and **B,** Summary of histological and molecular characterization of donor tumors (**A**) and PDX models (**B**). **C,** H&E staining of NSCLC PDXs. **D,** Engraftment success of primary NSCLCs and lung metastases considering the metabolic phenotype of the patient’s tumor. “High” and “low” TCA cycle enrichment was defined in Fig. 3B and C. **E,** Patient samples (lung, *n* = 64; primary NSCLC, *n* = 66) are plotted from Fig. 1C. Mice bearing NSCLC PDXs (mx57, *n* = 8; mx73, *n* = 6; mx95, *n* = 3; mx148, *n* = 3) were infused with [U-^13^C]glucose. Average TCA cycle enrichment was calculated and compared with primary NSCLC and adjacent lung tissue. Differences in enrichment were determined by a Kruskal–Wallis test with Dunn’s test. Data are expressed as average and SD. ADC, adenocarcinoma; NS, not statistically significant; Pleo, pleomorphic; SQCC, squamous cell carcinoma. Scale bar, 200 μm. ****, P<0.0001. NS, not significant.

We next compared the NSCLC PDXs with the original tumor (Fig. 4A and B). Histological analyses were performed by a board-certified pathologist. Samples were stained with hematoxylin and eosin (H&E), and IHC was performed for cytokeratin 7 (CK7), thyroid transcription factor-1 (TTF-1), and p40 (representative data in Fig. 4C and Supplementary Fig. S5C). All NSCLC PDXs matched the histological diagnosis of the patient’s tumor. Targeted sequencing panels revealed that all PDXs contained mutations observed in the patient’s tumor (Fig. 4A and B). Mx95 also contained a *PIK3CA* missense variant (H1047L) that was not detected in the patient because the variant was not on the panel used to assess that tumor. Likewise, the p53^Glu56fs^ variant detected in patient 148 was not included on the panel used to analyze the PDXs. A limited analysis was also performed on the lung metastases and PDXs generated from them (Supplementary Fig. S5D and S5E). Among the patients whose tumors were studied by ^13^C infusions, all but one from whom a PDX was established had high labeling of TCA cycle intermediates in the patient (Fig. 4D; Supplementary Fig. S5F). Overall, metastatic tumors established PDXs at a higher rate than NSCLCs (metastatic tumors: 6/9, 67%; NSCLC: 4/53, 7.5%).

It is unknown whether PDXs recapitulate the ^13^C labeling phenotypes of the original tumor. To address this, we infused [U-^13^C]glucose into mice bearing four subcutaneous NSCLC PDXs, using a tracing approach analogous to the one used in the patients (30). These experiments revealed that when considered as a group and normalized to labeling in glucose, TCA cycle labeling in the PDXs was indistinguishable from TCA cycle labeling in tumors from patients (Fig. 4E). Labeling features from all PDXs are in Supplementary Table S5. TCA cycle labeling in the PDXs exceeded labeling in nonmalignant lung tissue from the patients although it was similar to labeling in mouse lungs (Fig. 4E). This result reflects the much higher labeling in mouse than in the human lung under these conditions.

**Figure 5.**
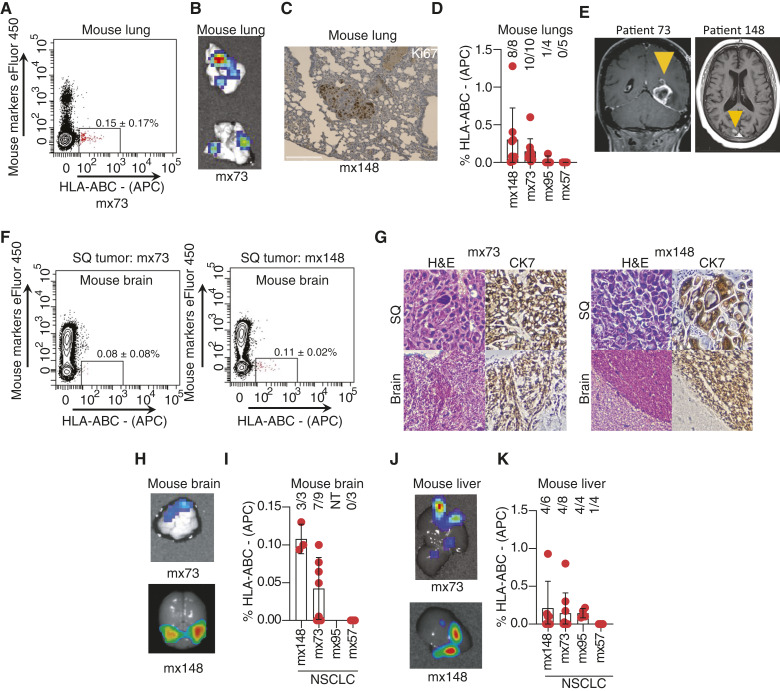
PDXs generated from primary NSCLCs spontaneously metastasize in NSG mice. **A,** Flow cytometry analysis of lung tissue from a mouse engrafted with mx73. Cells were stained with mouse lineage markers (CD45, CD31, and TER119) and HLA. **B,** Bioluminescence of mouse lungs bearing metastases from mx73. **C,** IHC staining for Ki67-positive cells in lung metastasis from mx148. **D,** Percentage of HLA-ABC–expressing cells detected in the lungs of mice bearing NSCLC PDXs. Each dot represents one mouse. Data are expressed as average and SD. The numerator and denominator reflect the number of mice with detectable HLA-ABC–expressing cells in the lung and the number of PDX-bearing mice analyzed, respectively. **E,** CT scans of brain metastases in patients 73 and 148. Yellow arrowheads indicate metastatic lesions. **F,** Flow cytometry analysis of mouse brains with metastatic cells from PDX mx73 (left) and PDX mx148 (right). **G,** H&E staining and IHC for CK7 in subcutaneous tumors and brains of mice bearing PDX mx73 (left) and PDX mx148 (right). **H,** Representative bioluminescence images of brains of mice with subcutaneous mx73 and mx148 PDXs. **I,** Summary of brain metastases observed in NSCLC PDXs. **J,** Representative bioluminescence images of liver metastases. **K,** Summary of liver metastasis in NSCLC PDXs. All data are expressed as average and SD. NT, not tested; SQ, subcutaneous.

To examine the metastatic capacity of the NSCLC PDXs, tumor cells were subcutaneously injected into NOD/SCID gamma (NSG) mice. The mice were euthanized when the tumor measured 2.0 cm in diameter, and organs were examined for metastases. In a mouse bearing PDX mx73, flow cytometry of a single-cell suspension from the lung revealed a population of cells expressing HLA (Fig. 5A). This population was not observed in non–PDX-bearing mice (Supplementary Fig. S6A). Bioluminescence imaging revealed luciferase-expressing cells in the lungs of mice with subcutaneous PDX tumors (Fig. 5B). In mice bearing PDX mx148, IHC analysis for Ki-67 confirmed the presence of small lesions with abundant proliferating cells (Fig. 5C). Metastases were additionally confirmed by H&E and NSCLC markers (Supplementary Fig. S6B). All NSCLC PDXs except for mx57 were capable of metastasizing to the lung, as assessed by the presence of viable, HLA-expressing cells (Fig. 5D).

Six patients in our cohort were diagnosed with brain metastasis, including two (patients 73 and 148, Fig. 5E) from whom we developed PDXs from the lung tumor. Flow cytometry on disaggregated cells from the brains of tumor-bearing (mx73 and mx148) mice revealed cells expressing HLA (Fig. 5F). A subset of these mice developed brain masses visible on histology and positive for expression of CK7, as in the subcutaneous PDXs (Fig. 5G), and some developed bioluminescence signal in their brains (Fig. 5H). Among the four new NSCLC PDX models, mice implanted with mx148 or mx73 usually had live human tumor cells in the brain. Mx95 was not tested, and mx57 did not develop brain metastases in the mice studied (Fig. 5I).

These PDXs also metastasized to other organs such as the liver, as observed by bioluminescence imaging or flow cytometry (Fig. 5J and K). Based on their consistent metastasis to multiple organs, mx73 and mx148 were analyzed further in subsequent experiments.

### Oxidative Phosphorylation Is a Metabolic Vulnerability for Metastasizing Cells

**Figure 6.**
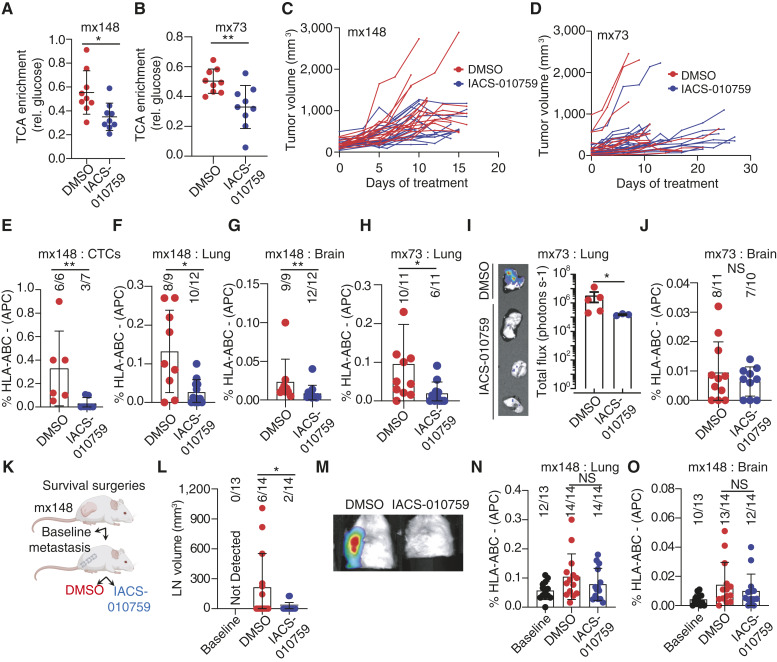
Inhibition of complex I with IACS-010759 limits metastasis in NSCLC PDXs. **A** and **B,** Tumor-bearing mice were treated daily with DMSO or IACS-010759 (5 mg/kg) by oral gavage for 3–4 weeks and then infused with [U-^13^C]glucose for 3 hours. Overall TCA cycle enrichment was calculated as the sum of isotopologues of citrate, malate, and glutamate and compared between DMSO- and IACS-010759–treated groups. **C** and **D,** Subcutaneous tumor volume calculated as (L × W^2^)/2. Each line represents the growth pattern of an individual tumor. **E,** Flow cytometry analysis of circulating tumor cells in the blood of mice engrafted with mx148. Cells were assessed for hCD45 and HLA. **F** and **G,** Lung and brain metastases from mx148 were evaluated by flow cytometry for mouse lineage markers and hCD45 and HLA. **H,** Metastatic burden in the lung from mx73 was assessed by flow cytometry, as above. **I,** Bioluminescence measurements of lung metastases. Representative BLI image (left) and quantitation (right) are displayed. **J,** Metastatic burden in the brain from mx73 was measured by flow cytometry. **K,** Schematic of the survival surgery approach to assess the impact of IACS-010759 on established metastases. **L,** Measurement of axillary lymph nodes containing macrometastases. **M,** Representative bioluminescence images of axillary lymph nodes. **N** and **O,** Metastatic burden in the lung (**N**) and brain (**O**) at the time of resection of the subcutaneous tumor (baseline) and after 2.5 weeks of treatment with DMSO or IACS-010759 subsequent to tumor resection. All data are expressed as average and SD. Statistical significance was assessed using Mann–Whitney or *t* tests to compare treatment groups. The number of mice tested and the number of mice with measurable metastases are indicated in each graph. CTCs, circulating tumor cells; LN, lymph node. *, P<0.05; **, P<0.01. NS, not significant.

The high TCA cycle enrichment in NSCLC tumors that progress (Fig. 3B–E) and in lung metastases (Fig. 1E) prompted us to ask whether suppressing TCA cycle metabolism suppresses metastasis. To test this, we treated mice with palpable NSCLC PDXs daily with IACS-010759, an ETC complex I inhibitor (31). The activity of the TCA cycle is usually coupled to the ETC and OXPHOS, in part because NADH generated by the TCA cycle is recycled to NAD^+^ by complex I. Accordingly, previous studies demonstrated that daily administration of 5 mg/kg IACS-010759 reduces [U-^13^C]glucose-derived labeling in the TCA cycle (32). Consistent with these findings, the drug suppressed labeling of TCA cycle intermediates in mx148 and mx73 PDXs (Fig. 6A and B). This dose of the drug was tolerated by the mice without inducing any changes in body weight (Supplementary Fig. S6C and S6D). The drug did not suppress the growth of the subcutaneous tumors (Fig. 6C and D). Nevertheless, by several metrics, IACS-010759 suppressed the metastatic spread of these tumors. In mx148, IACS-010759 reduced the number of viable human cancer cells in the circulation, lung, and brain (Fig. 6E–G; Supplementary Fig. S6E). In these experiments, most mice still had human cancer cells in the lung or brain, but the fraction of these cells was lower in IACS-010759–treated mice. Similar effects were also observed in mice implanted with mx73, in which IACS-010759 reduced metastatic burden in the lung (Fig. 6H and I). In this model, IACS-010759 modestly reduced the mean fraction of HLA-expressing cells in the brain although this did not reach statistical significance (Fig. 6J). These data demonstrate that NSCLC PDXs retain the ability to label TCA cycle intermediates with ^13^C originating on circulating glucose, that this activity can be blocked by a complex I inhibitor, and that complex I inhibition suppresses metastasis without impairing subcutaneous tumor growth.

To test if IACS-010759 is capable of reducing established metastases, we allowed subcutaneous tumors to reach a diameter of 1.0 to 1.5 cm in untreated mice and then resected the subcutaneous tumor to give existing metastases more time to grow (Fig. 6K). We quantified the metastatic burden in some mice at the time of surgery and allowed the rest to recover for 3 days before treating with DMSO or IACS-010759 for 2.5 weeks. At the time of surgery, no mice had lymph node metastases on gross inspection, but 6/14 DMSO-treated mice developed large axillary lymph node metastases during the therapeutic phase of the experiment. Only 2/14 IACS-010759–treated mice developed such metastases over this time frame, and lymph node volume was reduced in these mice (Fig. 6L; representative bioluminescence in Fig. 6M). The effects of IACS-010759 were less pronounced in other organs. There was a trend toward increased metastatic burden in the lung and brain between the surgery and endpoint analysis, but this was not affected by IACS-010759 (Fig. 6N and O).

## Discussion

Primary NSCLCs have extensive metabolic heterogeneity. In culture, even when propagated under identical conditions, cell lines derived from different NSCLCs display large cell-autonomous differences in nutrient utilization rates and the TCA cycle (33). *In vivo*, NSCLC tumors are variable in PET metrics of FDG uptake and oxidize multiple nutrients, with metabolic heterogeneity influenced by factors intrinsic and extrinsic to the cancer cells (21, 22, 26). Here, we provide evidence from a prospective, longitudinal cohort of patients subjected to intraoperative infusions with [U-^13^C]glucose that metabolic features correlate with clinical outcomes. This is remarkable because survival was largely dictated by metastatic cancer, and the isotope infusions were performed months to years before metastases were clinically apparent. The data indicate that heterogeneous metabolic properties of localized lung cancers in patients, reported by metabolite labeling from [U-^13^C]glucose, reflect biological capabilities associated with cancer progression.

Among the features we evaluated, labeling in TCA cycle intermediates best correlated with poor survival. Related metrics, particularly the abundance of TCA cycle intermediates, were not predictive. The lack of information provided by TCA cycle intermediate abundance demonstrates the value of using isotope tracers to assess the fuel sources that supply these metabolite pools. We note that the relationship between TCA cycle labeling and poor survival cuts across features such as histology and oncogenic driver mutations. We interpret this to mean that TCA cycle labeling is a convergent metabolic property of aggressive NSCLC, i.e., it arises within the context of histological and genomic heterogeneity but reflects a propensity for rapid progression and early demise. Maximum FDG-PET standardized uptake value correlates both with poor survival and with TCA cycle labeling in this cohort, indicating that as previously suggested (21, 22), oxidation in the TCA cycle is a fate of glucose-derived carbon in FDG-avid lung cancers.

Various cells in the tumor microenvironment import and process glucose. In mice, myeloid cells have been shown to consume glucose at a higher rate per cell than cancer cells (34). Therefore, we assume that myeloid and other cells contribute to TCA cycle labeling within human NSCLCs. However, single-cell RNA-seq data from two different human NSCLC studies indicate that within tumors, epithelial cells have OXPHOS expression scores as high or higher than any other cell type (27, 35). Although gene expression does not necessarily predict metabolic activity, these data are consistent with our observation that modulating oxidative metabolism within cancer cells is sufficient to affect tumor TCA cycle labeling (32).

TCA cycle labeling is low in benign lung lesions but high in lung metastases. This may indicate that the use of glucose and glucose-derived intermediates to fuel the TCA cycle is a generalized feature of aggressiveness. Evidence from multiple sources supports this idea. In mice, breast cancer metastases to the lung have increased TCA cycle flux compared with the primary orthotopic tumor (36). In melanoma, metastasis to the brain is associated with high OXPHOS gene expression, and the growth of melanomas implanted into the mouse brain can be suppressed by IACS-010759 (12). In human clear-cell renal cell carcinoma, direct analysis of tumor metabolism using [U-^13^C]glucose infusions reported higher labeling of TCA cycle intermediates from metastatic lesions than from primary kidney tumors (37). There are several possibilities for why metastatic tumors favor metabolic phenotypes involving enhanced TCA cycle labeling from [U-^13^C]glucose. One is that these tumors have adapted to thrive in organs with abundant oxygen, such as the lung and brain. This could promote oxidative metabolism, resulting in elevated labeling in the TCA cycle. Another is that enhanced oxidative metabolism provides an advantage to cancer cells during the metastatic cascade. Although these possibilities are not mutually exclusive, our data are most consistent with a role for OXPHOS during metastasis, prior to adaptation in the destination tissue, because IACS-010759 reduces the abundance of viable cancer cells in the circulation and because ETC inhibition after seeding distant organs seems not to reduce the ultimate metastatic burden, at least in these models.

A few limitations of the study should be considered. Our cohort consists primarily of White, non-Hispanic patients, and we do not know how this distribution affects tumor metabolic features. We did not account for some factors that can influence tumor metabolism, such as diet or circadian rhythm, although almost all patients were infused early in the morning after an overnight fast. ^13^C labeling of TCA cycle intermediates indicates oxidation of glucose-derived pyruvate, but this does not ensure that all metabolisms occur within the tumor, particularly the conversion of glucose to pyruvate. Some labeled pyruvate in the tumor likely arises from circulating ^13^C-lactate produced elsewhere in the body (21, 22). A related limitation is that we do not know if tumors with high TCA cycle labeling have simply undergone a shift toward glucose-derived pyruvate as a fuel for the TCA cycle or whether other substrates are also being oxidized to a greater extent. At a steady state, increased ^13^C labeling reflects an increased contribution of the labeled substrate to the metabolic product rather than an increase in the rate at which the product is produced (24). But in states of high OXPHOS and high TCA cycle turnover, multiple fuels may be providing carbon to the TCA cycle. This is particularly relevant to solid tumors, in which mixtures of cell types, chaotic vasculature, and inconsistent nutrient delivery produce regional metabolic heterogeneity, and probably means that not all cells within the fragment reach labeling steady state concurrently. This complicates the interpretation of the labeling data and makes it difficult to make assumptions that might apply to more homogeneous tissues.

Despite these limitations, multiple markers of TCA cycle labeling correlate with poor outcomes in patients, and partial ETC complex I blockade suppresses metastasis in PDXs derived from these same tumors. Understanding the basis of this dependence is worthy of further exploration. It is possible that complex I dependence reflects an increased need for efficient energy generation, as metastases are surmised to have higher metabolic demands than primary tumors in mice (36). But a functional ETC also allows cells to produce biosynthetic intermediates from the TCA cycle, maintain a favorable NAD^+^/NADH ratio, mitigate oxidative stress, maintain a mitochondrial membrane potential for protein and solute transport, and generate other signals that promote growth. Prolonged, systemic complex I blockade with IACS-010759 induces lactic acidosis and neurotoxicity in patients (38), but perhaps a more tailored approach aimed at the specific advantages provided by OXPHOS to metastasizing cells would be better tolerated. It is also worth noting that the association between OXPHOS and cancer progression extends beyond metastasis, with various models of tumor relapse in mice also converging upon an increased need for this pathway (39, 40). Therefore, despite the ubiquity of glycolysis in malignant cells, multiple forms of cancer progression converge upon an increased need for OXPHOS.

## Methods

### Lead Contacts

Further information and requests for resources and reagents should be directed to and will be fulfilled by the Lead Contact(s) Ralph DeBerardinis (Ralph.DeBerardinis@UTSouthwestern.edu) or Brandon Faubert (bfaubert@uchicago.edu).

### Experimental Model and Study Participant Details

#### Human Subjects

A total of 143 patients with a suspicious lung lesion were enrolled in an institutional review board–approved protocol after obtaining written informed consent (NCT02095808). Studies were conducted in accordance with recognized ethical guidelines (U.S. Common Rule). Patients were considered eligible for the study if they had surgically resectable pulmonary masses. Uncontrolled diabetes was a contraindication to participation in ^13^C glucose infusion. Clinical details about patients participating in the study and their tumors are summarized in Supplementary Table S1.

#### Animal Studies

All procedures were approved by UT Southwestern’s (2017-102008) and the University of Chicago’s (72678) Institutional Animal Care and Use protocols. Healthy male or female NSG mice were xenografted at 8 to 10 weeks of age. All mice were housed in pathogen-free conditions prior to use. Prior to the described studies, mice were monitored regularly and determined to be healthy by the veterinary staff. Tumor cell suspensions were prepared for injection in Hank’s Balanced Salt Solution (HBSS), with 50% Matrigel (BD Biosciences, 354248) or Cultrex Basement Membrane (R&D, 343201001). For established PDX lines, subcutaneous injections were performed in the right flank, transplanting 5 to 10 × 10^5^ cells. Mouse cages were randomized between treatments, and mice within the same cage underwent the same treatment. Subcutaneous tumors were measured with calipers 2 to 3 times per week after the onset of tumor growth, until tumors reached 2.0 cm in diameter or mouse body score was 2.5 or less. At this point, mice were infused with ^13^C glucose or euthanized and analyzed for spontaneous metastasis by bioluminescence or flow cytometry.

### Method Details

Product numbers and available research resource identifiers (RRIDs) are provided in Supplementary Table S6.

#### Patient Infusions

All patients were fasted overnight prior to the surgery, and most surgeries were performed the following morning, usually at 7:00 am. Before the start of surgery, a peripheral intravenous line was placed. A bolus (8 g over 10 minutes) of sterile, pyrogen-free [U-^13^C]glucose (Cambridge Isotope Laboratories or Isotec) was administered, followed by 4 or 8 g/hour infusion for the duration of the surgery (generally for at least 2 hours prior to tumor resection). Blood samples were drawn to analyze ^13^C enrichment either from a second peripheral intravenous line in the contralateral arm or from an arterial line placed by the anesthesia team. In all cases, standard surgical procedures were followed, with the majority of cases being robotic lobectomies. Preoperative imaging and gross inspection at resection were used to sample viable fragments of the tumor and lung. In cases in which metabolic heterogeneity was assessed, the resected lobe was oriented anatomically to identify regions of the tumor preselected by imaging. Tissue fragments were rinsed in ice-cold saline and immediately frozen in liquid nitrogen. If the tissue sample was of adequate size, a subsection was cut and placed in ice-cold HBSS for PDX generation. Two patients were infused twice. Patient K1050 underwent an infusion during resection of their primary NSCLC. This patient later presented with another pulmonary lesion and was infused again (K1124). Because we could not determine whether this second lesion was another primary NSCLC or an intrathoracic metastasis, we did not include this patient in the survival analysis. Similarly, a patient presented with metastatic lung lesions on two separate occasions and was also infused twice (K1170 and K1203).

#### IHC

Tissues were fixed in 4% paraformaldehyde overnight at 4°C, washed in PBS, and stored at 4°C. Serial sections (4 μm thickness) were prepared from formalin-fixed, paraffin-embedded lung samples. Standard H&E staining was performed for tissue morphology visualization. Tissue slides were stained for NSCLC markers (CK7, TTF-1, and p40) after quenching endogenous peroxidase activity. Slides were incubated with anti-CK7 antibody (RN7, Cell Signaling Technology), anti-TTF-1 (Cell Signaling Technology), and anti-p40 (Cell Signaling Technology) and were all incubated at 1:250 overnight in a humid chamber at 4°C. Slides were washed with TBS/T 3 times before incubation with antimouse secondary for 1 hour at room temperature. Antigens were revealed with the Polymer Detection Kit with peroxidase (MP-7422-15). Slides were analyzed using Aperio ImageScope software.

#### Mutation Analysis

Patient’s DNA was isolated from blood or formalin-fixed, paraffin-embedded specimens after microdissection. Most analyses used a 50-gene clinical next-generation sequencing assay, the AmpliSeq Cancer Hotspot Panel v2 run on an Ion Torrent Personal Genome Instrument. Software included Torrent Variant Caller, Integrative Genomics Viewer, and Geneticist Assistant software. In some cases, mutational analyses were performed either with commercial testing (Guardant 360) or were performed offsite with a different gene panel.

#### IHC Analyses of Macrophage Content

IHC staining for CD68 was used to generate H-scores for macrophages from 18 tumor tissue slides. Additionally, immune infiltrates were deconvoluted from RNA-seq data of 45 patients using TIMER2.0 (41). IHC images were further deconvoluted into hematoxylin and 3,3′-diaminobenzidine tetrahydrochloride (DAB) channels to separate nuclear staining from CD68-positive signals. StarDist (42–44) was applied to segment nuclei in the hematoxylin channel, followed by a five-pixel extension around each nucleus to define a cytoplasmic region, which was used to quantify DAB staining. Cells were classified as CD68-positive based on the percentage of DAB-positive pixels within this extended region.

#### Generating PDXs

Tumor tissue samples obtained in the operating room were placed in HBSS on ice. The tissue was minced using a scalpel and placed in a tissue digestion buffer of HBSS (Thermo Fisher Scientific), Collagenase IV (Cell Stem Cell), and CaCl_2_ (Sigma) for 20 minutes at 37°C. Samples were filtered through a 70 micron filter and centrifuged for 5 minutes at 400 *g*. Cells were resuspended in HBSS, mixed 1:1 with Matrigel (Becton Dickinson), and then subcutaneously injected into the flanks of immunodeficient mice (NSG). The mice were monitored for up to 1 year after injection for tumor growth. When the tumor diameter reached ∼1.5 cm, the tumors were excised and digested to a single-cell suspension. New tumors were established by implanting 5 to 10 × 10^5^ cells into NSG mice. The remaining cells were aliquoted for storage in liquid nitrogen. After engraftment, the mice were monitored after each passage for the presence of B-cell lymphoma by flow cytometry, after staining for hCD45 and HLA antibodies. When B-cell lymphoma cells were detected, the PDX samples were depleted of these cells by FACS (detailed in “Cell Labeling and Flow Cytometry”). In brief, dissociated tumors were stained with HLA-ABC and hCD45-FITC, and live, CD45-negative cells were obtained by FACS for reinjection.

#### Lentiviral Transduction of PDXs

The virus was produced by combining 0.9 μg of the dsRED–luciferase plasmid with 1 μg of helper plasmids (0.4 μg MD2G and 0.6 μg psPAX2) and transfecting into 293T cells using lipofectamine (Thermo Fisher Scientific). The viral supernatants were collected 48 hours after transfection and filtered through a 0.45-micron filter. Freshly dissociated NSCLC cells were transduced with viral supernatants supplemented with 10 μg/mL polybrene (Sigma) for 8 hours. Cells were washed twice with HBSS, and approximately 1 × 10^5^ cells per mouse were suspended in HBSS, mixed 1:1 with Matrigel, and injected subcutaneously into NSG mice. After growing to 1 to 1.5 cm in diameter, tumors were excised and dissociated as above, and dsRED-positive cells were sorted by flow cytometry and reinjected into the flanks of new NSG mice.

#### PDX Mouse Infusions

Infusions occurred when tumors were <1.5 cm in diameter. Between 9:00 and 10:00 am, 25-gauge catheters were placed in the lateral tail vein under anesthesia with ketamine/xylazine. Isotope infusions started immediately after implantation of the catheter and continued for approximately 3 hours, also under anesthesia. In the [U-^13^C]glucose infusions, the total dose of glucose was 2.48 g/kg dissolved in 750 μL saline. The glucose solution was administered as a bolus of 125 μL/minute (1 minute) followed by a continuous rate of 2.5 μL/minute for 3 hours. Blood samples of ∼20 μL were obtained every 30 minutes via retro-orbital bleed. Animals were euthanized at the end of the infusion; then tumors were harvested, rinsed briefly in cold saline, and frozen in liquid nitrogen.

#### PDX Treatment and Sample Collection

Once tumors were palpable, the mice were treated daily with DMSO or IACS-010759 (5 mg/kg) in 100 μL 0.5% promethylcellulose, 0.2% Tween80, and 5% DMSO by oral gavage, as adapted from ref. 31. At the end of treatment, circulating tumor cells (obtained by cardiac puncture) or metastatic cells were harvested. Circulating tumor cell preparations underwent red blood cell lysis (Thermo Fisher Scientific) before flow cytometry analysis. Tissue samples (subcutaneous tumor and organs) were rinsed in saline, cut, and immediately snap frozen in liquid nitrogen, fixed in 4% paraformaldehyde, or digested into a single-cell suspension as described above.

#### Surgical Removal of the Subcutaneous Tumor

When subcutaneous tumors reached 1.0 to 1.5 cm in diameter, the tumor was surgically resected. The mice were anesthetized with isoflurane (0.5%–4% for induction and 2%–3% for maintenance). The skin covering the tumor was cleared of hair, and the surgical area was sterilized with three alternating scrubs of betadine and 70% alcohol. An incision was made across the skin to expose the tumor. Using sterilized scissors and forceps, the tumor was excised from the subcutaneous space. The wound was then closed with surgical clips, and the mice were dosed with buprenorphine slow-release (0.5–1.0 mg/kg) every 48 to 72 hours. The wound clips were removed 10 to 12 days later.

#### Bioluminescence Imaging

Ten minutes prior to imaging, mice were injected intraperitoneally with 100 μL of D-luciferin monopotassium salt (40 mg/mL) in PBS. Mice were anesthetized with isoflurane and imaged using an IVIS Imaging System 200 Series (Caliper Life Sciences) with Living Image software. Mice were euthanized, and individual organs were surgically removed and imaged. The exposure time ranged from 10 to 60 seconds, depending on maximal signal intensity to avoid oversaturation of the signal. Background luminescence was assessed with a non–tumor-bearing negative control mouse. Total photo flux was quantified with “region of interest” measurement tools in Living Image (PerkinElmer). Metastatic burden was calculated as the observed total flux in tumor-bearing mice minus the background flux of negative control mice.

#### Cell Labeling and Flow Cytometry

All antibody staining was performed for 20 minutes on ice, followed by washing with HBSS and centrifugation at 300 *g* for 5 minutes. Cells were stained with conjugated antibodies against mouse CD45 (eFluor 450, Thermo Fisher Scientific), mouse CD31 (eFluor 450, Thermo Fisher Scientific), mouse Ter119 (eFluor 450, Thermo Fisher Scientific), and HLA-ABC (APC, BioLegend). Cells were tested for B-cell lymphoma contamination by staining with hCD45 (FITC, BD Biosciences). Cells were examined on an LSRFortessa cell analyzer (BD Biosciences) or LSRII 4-12 (BD Biosciences). Cell sorting was performed on a FACS Fusion Cell Sorter (BD Biosciences). For analysis of circulating NSCLC cells, blood was collected from mice by cardiac puncture with a syringe pretreated with citrate–dextrose solution (Sigma). Red blood cells were lysed and removed prior to flow cytometry as previously described (45).

#### Gas Chromatography–Mass Spectroscopy

Blood was obtained prior to and approximately every 30 minutes during infusion until tissue was removed from the patient or mouse. Whole blood was chilled on ice and centrifuged to separate and freeze the plasma. Aliquots of 25 to 50 μL of plasma were added to 80:20 methanol/water for extraction. Frozen tissue fragments weighing 5 to 15 mg were added to 80:20 methanol/water and extracted to analyze ^13^C enrichment. Samples were subjected to three freeze-thaw cycles and then centrifuged at 16,000× *g* for 15 minutes to precipitate macromolecules. The supernatants were evaporated, then resuspended in 40 μL anhydrous pyridine containing methoxyamine (10 mg/mL), and added to preprepared gas chromatography–mass spectroscopy autoinjector vials containing 80 μL N-(*tert*-butyldimethylsilyl)-N-methyltrifluoroacetamide derivatization reagent. The samples were incubated at 70°C for 1 hour; then aliquots of 1 μL were injected for analysis. Each sample was injected in triplicate to confirm technical reproducibility. Samples were analyzed using either an Agilent 6890 or 7890 gas chromatograph coupled to an Agilent 5973N or 5975C Mass Selective Detector, respectively. The observed distributions of mass isotopologues were corrected for natural abundance.

#### RNA Sequencing

Tissue samples (adjacent lung or tumor) were homogenized, and RNA was extracted; 3 μg of RNA was processed for library preparation and sequencing, and raw reads were aligned to human genome assembly GRCh38. RNA-seq data were collapsed to the patient level by averaging data from multiple fragments of the same patient’s tumor. We used the REACTOME_RESPIRATORY_ELECTRON_TRANSPORT gene set from the MSigDB c2.cp Reactome gene set library. The OXPHOS score was computed using single-sample gene set enrichment analysis, implemented through the Gene Set Variation Analysis package in R. The single-sample gene set enrichment analysis scores represent the degree to which the genes in the set are coordinately upregulated or downregulated within a sample. This score can be used to compare the activity of this gene set across different samples or conditions and can provide insights into the biological processes that are active in each sample (46).

#### Quantification and Statistical Analysis

Patient-matched tumor and noncancerous lung samples, as well as intratumoral samples processed on the same day, were analyzed as described in the figure legends. All differences were considered significant if *P* < 0.05. Statistics were calculated using Prism software. Sample sizes were not predetermined based on statistical power calculations. Formal randomizations of mouse experiments were not used. Mice were allocated to experiments randomly and processed in arbitrary order. Data were tested for normal distribution and similar variance among treatments using the Shapiro–Wilk tests when 3 ≤ *n* < 20 or D’Agostino-Pearson omnibus tests when *n* ≥ 20. When the data significantly deviated from normality (*P* < 0.01) or variability significantly differed among treatments (*P* < 0.05), we log_2_-transformed the data and tested again for normality and variability. If the transformed data no longer significantly deviated from normality and equal variability, we performed parametric tests on the transformed data. If log_2_-transformation was not possible or the transformed data still significantly deviated from normality or equal variability, we performed nonparametric tests on the nontransformed data. To assess the statistical significance of a difference between the two groups, we used Student *t* tests or paired *t* tests (when a parametric test was appropriate), Welch’s *t* tests (when data were normally distributed but not equally variable), or Mann–Whitney or Wilcoxon tests (when a nonparametric test was appropriate).

### Data Availability

Source data for Figs. 1–6 and Supplementary Figs. S1–S6 are provided with the article. All other data are available from the corresponding authors upon request. RNA-seq data have been deposited at Database of Genotypes and Phenotypes (study accession: phs003880.v1.p1) and are publicly available as of the date of publication.

## Supplementary Material

Supplementary Table 1 (Related to Figure 1). Clinical and pathological data from patients recruited to this study.

Supplementary Table 2 (Related to Figure 1). Patient Isotopologue Data.

Supplementary Table 3 (Related to Figure 2). Transcriptomic data for NSCLC and adjacent lung tissue fragments.

Supplementary Table 4 (Related to Figure 3). Overall and recurrence-free survival summary.

Supplementary Table 5 Related to Figures 4 and 6). PDX Isotopologue Data.

Supplementary Table 6 Key Resources

Supplementary Figure 1 (Related to Figure 1). TCA cycle labeling and metabolite abundance in tumors and lungs from NSCLC patents.

Supplementary Figure 2 (Related to Figure 2). Epithelial and myeloid cell contributions to gene expression and 13C labeling features.

Supplementary Figure 3 (Related to Figure 3). Relationships between TCA cycle labeling and clinical factors.

Supplementary Figure 4 (Related to Figure 3). TCA cycle metabolite abundance does not correlate with overall survival.

Supplementary Figure 5 (Related to Figure 4). Development of patient-derived xenografts from malignant tumors in the lung.

Supplementary Figure 6 (Related to Figures 5 and 6): Treatment with IACS-010759 reduces distant metastasis.
