# Supplementary material for: High Glucose Contribution to the TCA Cycle Is a Feature of Aggressive Non–Small Cell Lung Cancer in Patients
Source: Cancer Discov. 2025 Feb 17;15(4):702–16. doi: 10.1158/2159-8290.CD-23-1319 (PMC11962397; doi:10.1158/2159-8290.CD-23-1319)
Supplement: Supplementary Figure 1 — (Related to Figure 1). TCA cycle labeling and metabolite abundance in tumors and lungs from NSCLC patents. [file cd-23-1319_supplementary_figure_1_suppsf1.pdf]

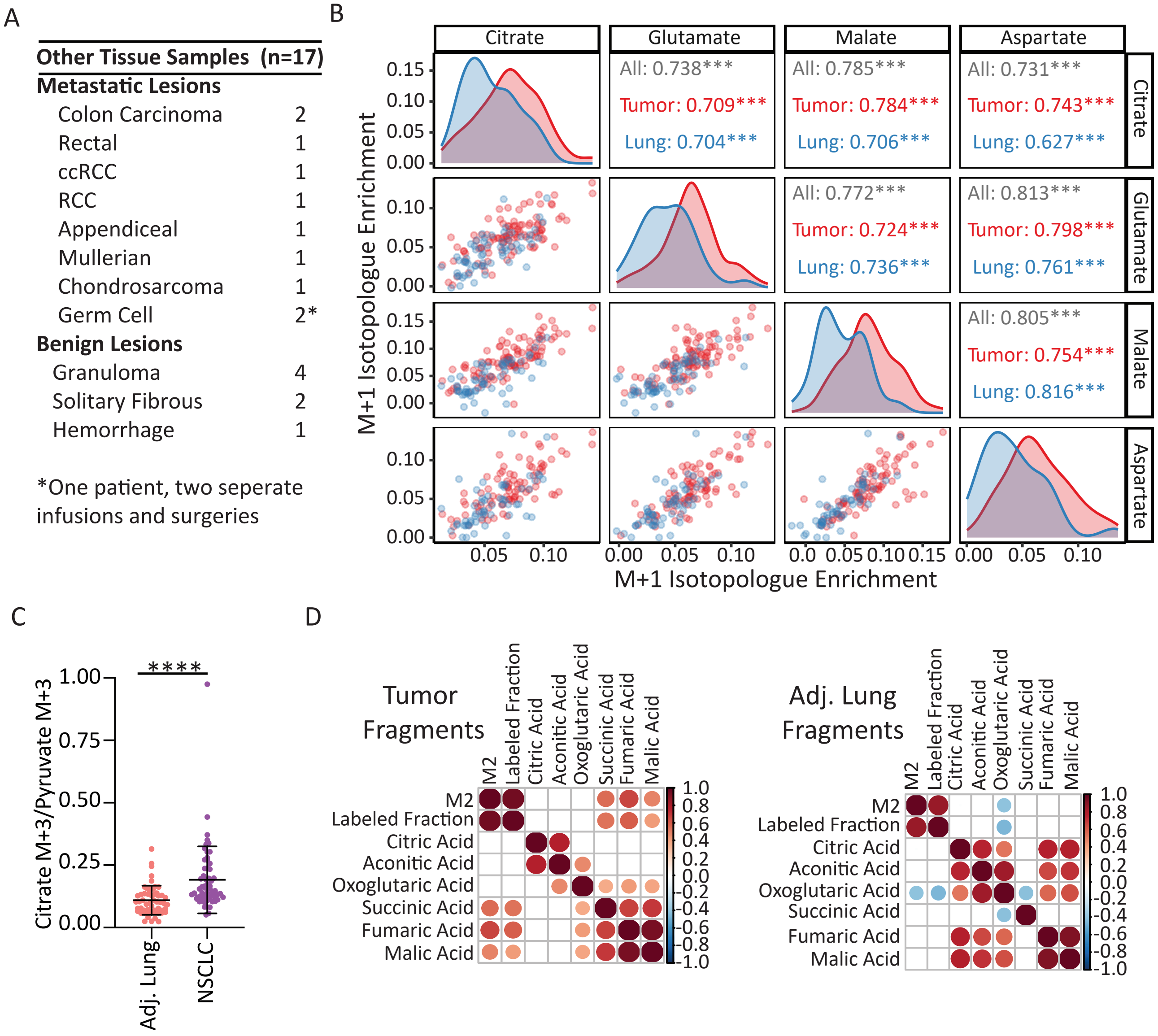

Supplementary Figure 1 (related to Figure 1). TCA cycle labeling and metabolite abundance in tumors and lungs from NSCLC patients. A) Histological types of metastatic and benign lesions. B) Spearman correlation analysis of ^{13}C enrichment (M+1) values between TCA cycle metabolites in each tissue. Values from each sample (blue: lung; red: NSCLC tumor) are plotted to compare labeling between each pair of metabolites. C) M+3 labeling in citrate normalized to pyruvate m+3 in adjacent lung and tumor tissue. D) Spearman correlation plots between labeling metrics and metabolite abundances are shown for tumor fragments (*left*) and adjacent lung (*right*). The averaged TCA enrichment for all isotopologues is referred to as 'labeled fraction'.
