# Supplementary material for: High Glucose Contribution to the TCA Cycle Is a Feature of Aggressive Non–Small Cell Lung Cancer in Patients
Source: Cancer Discov. 2025 Feb 17;15(4):702–16. doi: 10.1158/2159-8290.CD-23-1319 (PMC11962397; doi:10.1158/2159-8290.CD-23-1319)
Supplement: Supplementary Figure 2 — (Related to Figure 2). Epithelial and myeloid cell contributions to gene expression and 13C labeling features. [file cd-23-1319_supplementary_figure_2_suppsf2.pdf]

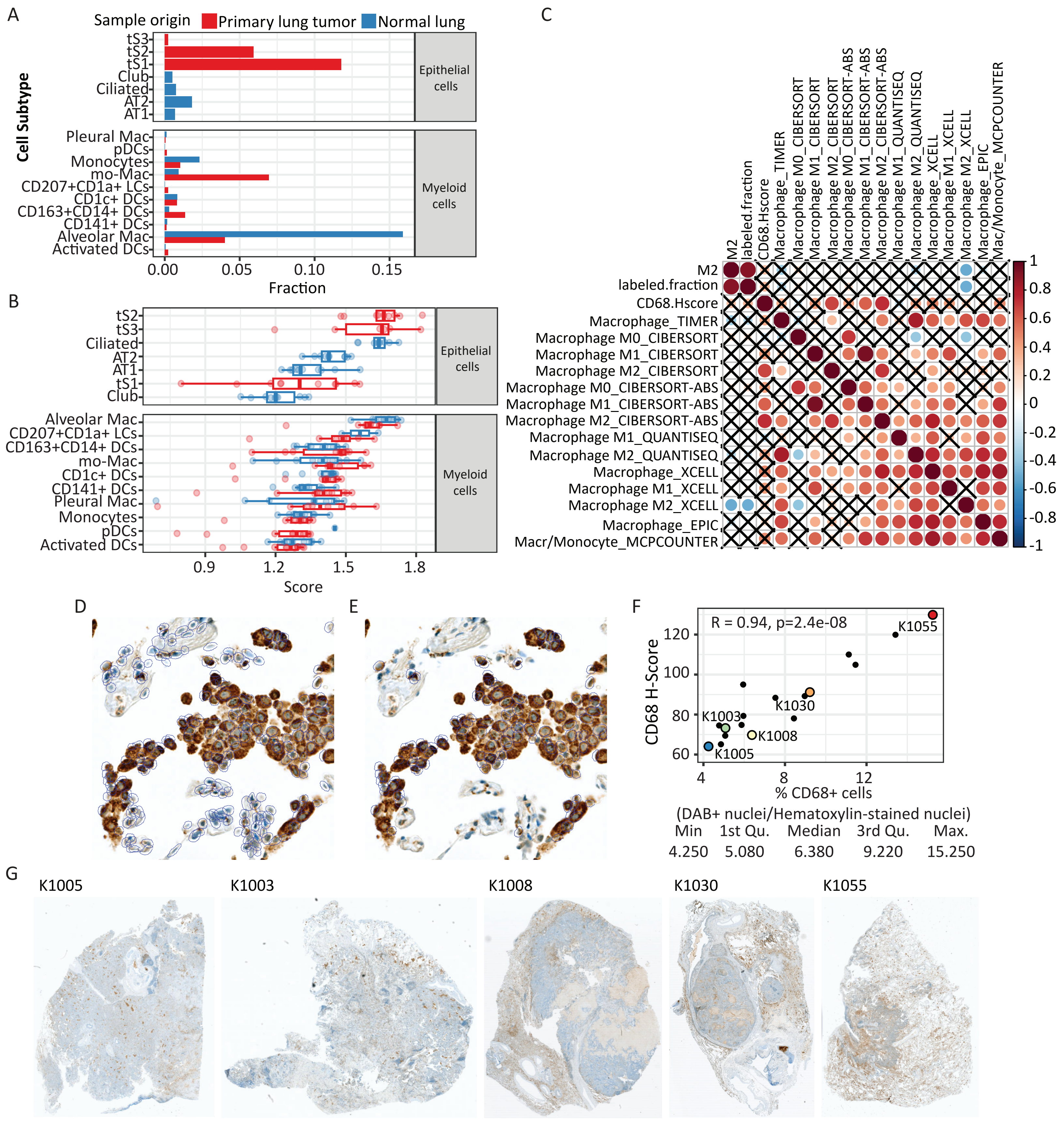

Supplementary Figure 2 (Related to Figure 2). Epithelial and myeloid cell contributions to gene expression and ^{13}C labeling features. A-B) Composition (A) and OXPHOS scores (B) of epithelial and myeloid cell subtypes in the Kim_2020 data. C) Pairwise Spearman correlation among TCA cycle labeling measurements (M2 and labeled fraction), IHC quantification (CD68 H-score), and transcriptome-deconvoluted macrophage estimates. Statistically insignificant correlations were crossed out. D) All nuclei segmented from the Hematoxylin channel. E) Nuclei from CD68+ cells. F) Comparison of CD68+ cell content from automated detection (x-axis) and CD68 H-score. Summary statistics at the bottom show the minimum, first quartile, median, third quartile, and maximum percentage of CD68-positive cells in the analyzed tissues. G) Low-magnification images of the CD68-stained IHC samples labeled in the scatter plot.
