# Supplementary material for: High Glucose Contribution to the TCA Cycle Is a Feature of Aggressive Non–Small Cell Lung Cancer in Patients
Source: Cancer Discov. 2025 Feb 17;15(4):702–16. doi: 10.1158/2159-8290.CD-23-1319 (PMC11962397; doi:10.1158/2159-8290.CD-23-1319)
Supplement: Supplementary Figure 3 — (Related to Figure 3). Relationships between TCA cycle labeling and clinical factors. [file cd-23-1319_supplementary_figure_3_suppsf3.pdf]

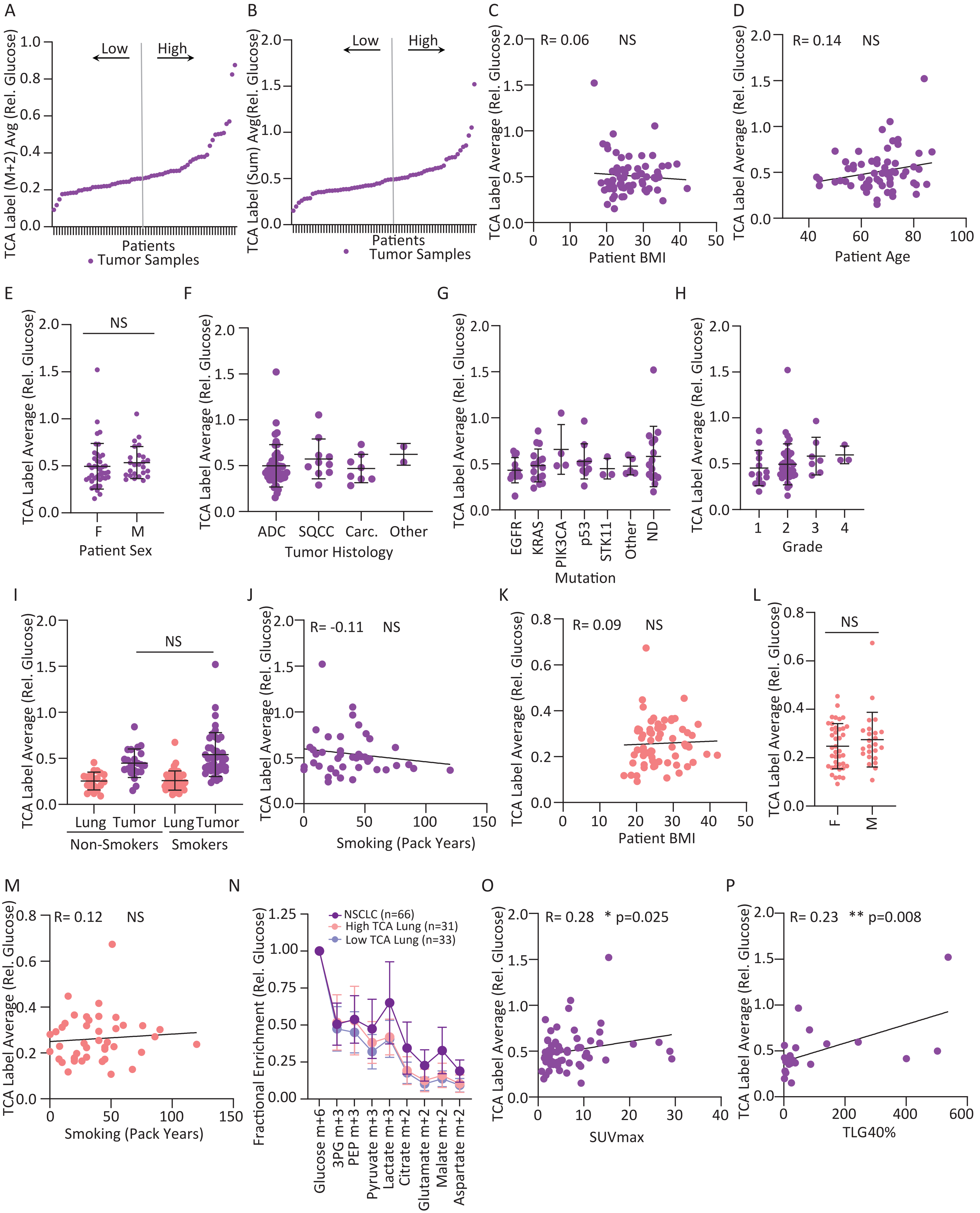
A
TCA Label (M+2) Avg (Rel. Glucose)
Low
High
Patients
Tumor Samples
B
TCA Label (Sum) Avg(Rel. Glucose)
C
R= 0.06 NS
TCA Label Average (Rel. Glucose)
Patient BMI
D
R= 0.14 NS
Patient Age
E
NS
Patient Sex
F
M
F
ADC
SQCC
Carc.
Other
Tumor Histology
G
EGFR
KRAS
PIK3CA
p53
STK11
Other
ND
Mutation
H
Grade
I
NS
Lung
Tumor
Non-Smokers
Smokers
J
R= -0.11 NS
Smoking (Pack Years)
K
R= 0.09 NS
L
M
R= 0.12 NS
N
Fractional Enrichment (Rel. Glucose)
NSCLC (n=66)
High TCA Lung (n=31)
Low TCA Lung (n=33)
Glucose m+6
3PG m+3
PEP m+3
Pyruvate m+3
Lactate m+3
Citrate m+2
Glutamate m+2
Malate m+2
Aspartate m+2
O
R= 0.28 * p=0.025
SUVmax
P
R= 0.23 ** p=0.008
TLG40%

Supplementary Figure 3 (related to Figure 3). Relationships between TCA cycle labeling and clinical factors. A) Tumor distribution based on average M+2 values of citrate, malate and glutamate, relative to glucose m+6. B) Tumor distribution based on the average of the sum of isotopologues of citrate, malate and glutamate (relative to glucose). C-E) Correlation analysis between TCA cycle labeling and patient body mass index (BMI) (C) age (D) and sex (E). F-H) TCA cycle labeling in tumor subsets defined by predominant histological type (F), mutation status (G) and grade (H). EGFR, KRAS and PIK3CA include tumors with or without a co-mutation of TP53 or STK11. Tumors under TP53 or STK11 have mutations in these genes but lack detectable mutations in EGFR, KRAS or PIK3CA. I) TCA cycle labeling of adjacent lung and tumor tissues of non-smokers (n=22) and patients who smoked (n=44). J) Correlation between TCA cycle labeling and pack-year smoking history (n=43). K-M) Correlation between TCA cycle labeling in adjacent lung tissue and patient features, such as (BMI) (K), sex (L) and smoking history (M). N) 13C enrichment in NSCLCs and in adjacent lung samples dichotomized according to whether the tumor had high or low TCA cycle labeling. Fractional enrichments of glycolytic (m+3) and TCA cycle (m+2) metabolites are normalized to enrichment of glucose (m+6) within the tissue. O) Correlation between TCA cycle labeling and SUVmax derived from ^{18}FDG-PET (n=66). P) Correlation between TCA cycle labeling and total lesion glycolysis (40%), a volumetric-based assessment of glucose accumulation from FDG-PET (n=29). Data are expressed as average and s.d. For all graphs, each dot represents 1 patient. Spearman correlations were defined as significant if $p<0.05$. Overall and recurrence-free survival were assessed by the log-rank (Mantel-Cox) test. Statistical significance was assessed using t-tests to compare tissue types. Multiple comparisons were adjusted using the Holm-Sidak's method.
