# Supplementary material for: High Glucose Contribution to the TCA Cycle Is a Feature of Aggressive Non–Small Cell Lung Cancer in Patients
Source: Cancer Discov. 2025 Feb 17;15(4):702–16. doi: 10.1158/2159-8290.CD-23-1319 (PMC11962397; doi:10.1158/2159-8290.CD-23-1319)
Supplement: Supplementary Figure 4 — (Related to Figure 3). TCA cycle metabolite abundance does not correlate with overall survival. [file cd-23-1319_supplementary_figure_4_suppsf4.pdf]

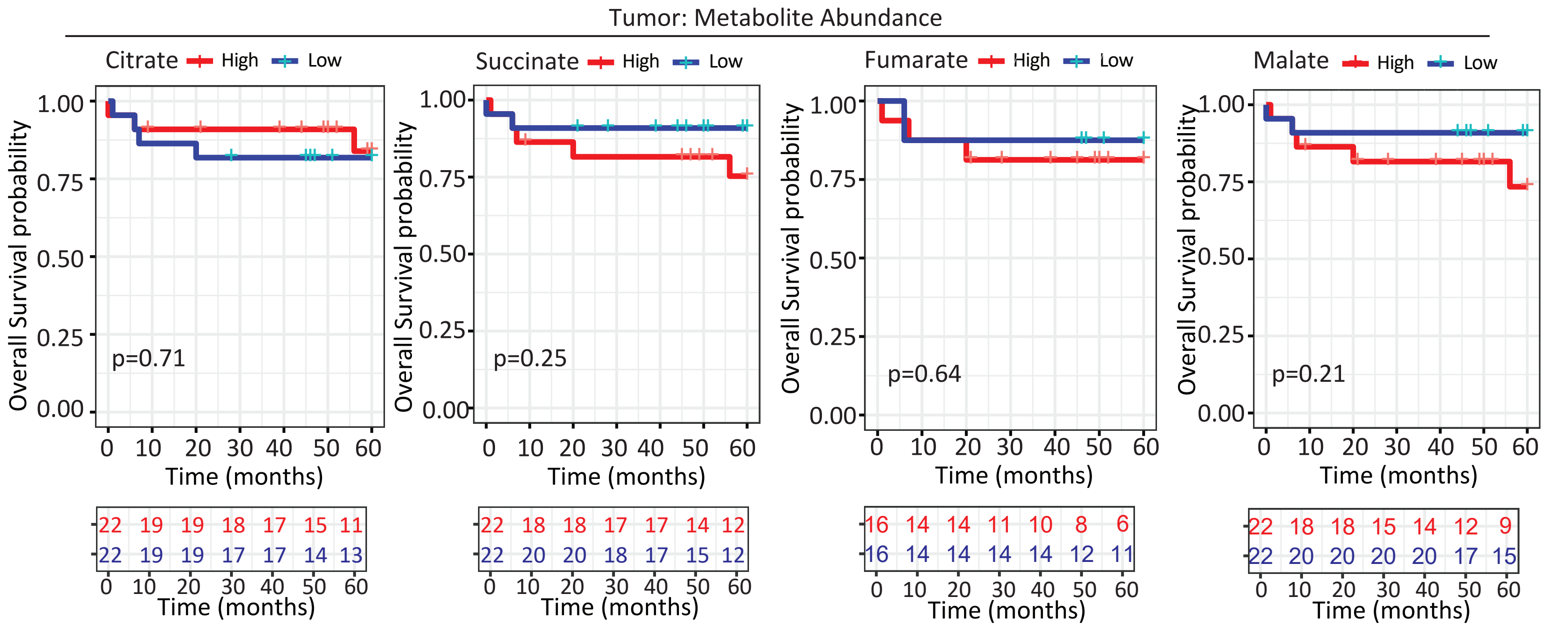

Supplementary Figure 4 (related to Figure 3). TCA cycle metabolite abundance does not correlate with overall survival. The relative abundances of citrate, succinate, fumarate and malate as assessed by metabolomics do not correlate with overall survival. The groups were determined by the top and bottom half of abundance values for each metabolite. Survival curves were assessed by the Log-rank (Mantel-Cox) test.
