# Supplementary material for: High Glucose Contribution to the TCA Cycle Is a Feature of Aggressive Non–Small Cell Lung Cancer in Patients
Source: Cancer Discov. 2025 Feb 17;15(4):702–16. doi: 10.1158/2159-8290.CD-23-1319 (PMC11962397; doi:10.1158/2159-8290.CD-23-1319)
Supplement: Supplementary Figure 5 — (Related to Figure 4). Development of patient-derived xenografts from malignant tumors in the lung. [file cd-23-1319_supplementary_figure_5_suppsf5.pdf]

A

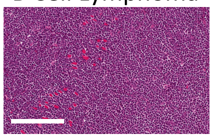

B

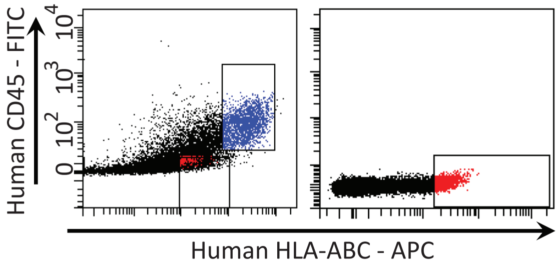

C

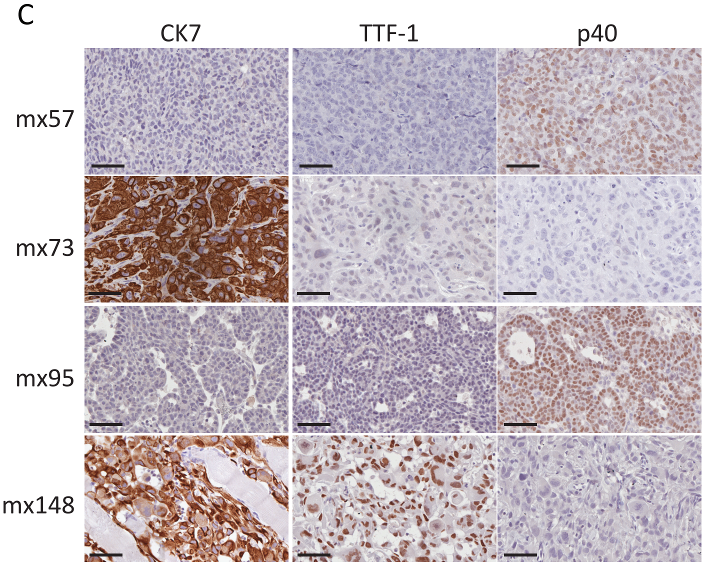

D

Tumor	Type	Pt. Tumor Mutation
108A/L	Colon	$KRAS^{G12V}$
133	Appendiceal	$ARID1B^{Q1818}$, $RUNX1T1^{R395W}$, $p53^{R306*}$
136	Mullerian	$p53^{H193R}$
161	Colon	$KRAS^{G12A}$, $PTEN^{R130G}$
182	Rectal	$p53^{R175H}$

E

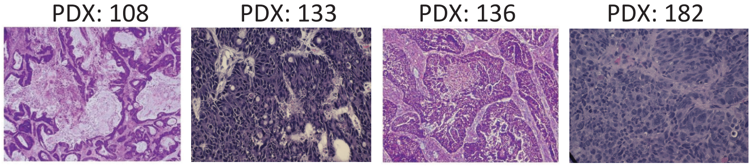

F

Tumor	TCA Label	Success/Total
NSCLC	High	2/20
	Low	0/12
	NA	2/21
Metastasis	High	5/5
	Low	1/3
	NA	0/1

Supplementary Figure 5 (related to Figure 4). Development of patient-derived xenografts from malignant tumors in the lung. A) Representative H&E of a B cell lymphoma generated during attempted establishment of an NSCLC PDX. Scale bar is 200µm. B) Flow cytometry plot of a NSCLC PDX contaminated with B cell lymphoma. HLA-positive/CD45 negative (red, NSCLC), and double-positive cell populations (blue, lymphoma) from the tumor in panel A. Contaminated tumors underwent flow cytometry cell sorting for HLA-positive, CD45-negative cells that were re-injected into NSG mice. PDX purity was assessed in established tumors (right). C) Representative IHC stains for NSCLC subtyping. MX57 was classified as an invasive squamous carcinoma in a lepidic pattern (CK7 negative; TTF-1 negative; p40 positive (nuclear). MX73 is a near-solid adenocarcinoma, with muscular and lymphovascular invasion (CK7 positive; TTF-1 weakly positive (nuclear); p40 negative). MX95 is an adenocarcinoma in a lepidic pattern (CK7 negative; TTF-1 negative; p40 positive). MX148 is an invasive carcinoma with pleomorphic cells with muscular invasion (CK7 positive; TTF-1 positive (nuclear), p40 negative). Scale bar is 60µm. D) Histological and molecular summary of metastatic patient tumors. E) H&E staining of PDXs generated from metastatic lesions. F) Fraction of successful PDX engraftment based on tumor type and TCA cycle labeling pattern.
