# Supplementary material for: High Glucose Contribution to the TCA Cycle Is a Feature of Aggressive Non–Small Cell Lung Cancer in Patients
Source: Cancer Discov. 2025 Feb 17;15(4):702–16. doi: 10.1158/2159-8290.CD-23-1319 (PMC11962397; doi:10.1158/2159-8290.CD-23-1319)
Supplement: Supplementary Figure 6 — (Related to Figures 5 and 6): Treatment with IACS-010759 reduces distant metastasis. [file cd-23-1319_supplementary_figure_6_suppsf6.pdf]

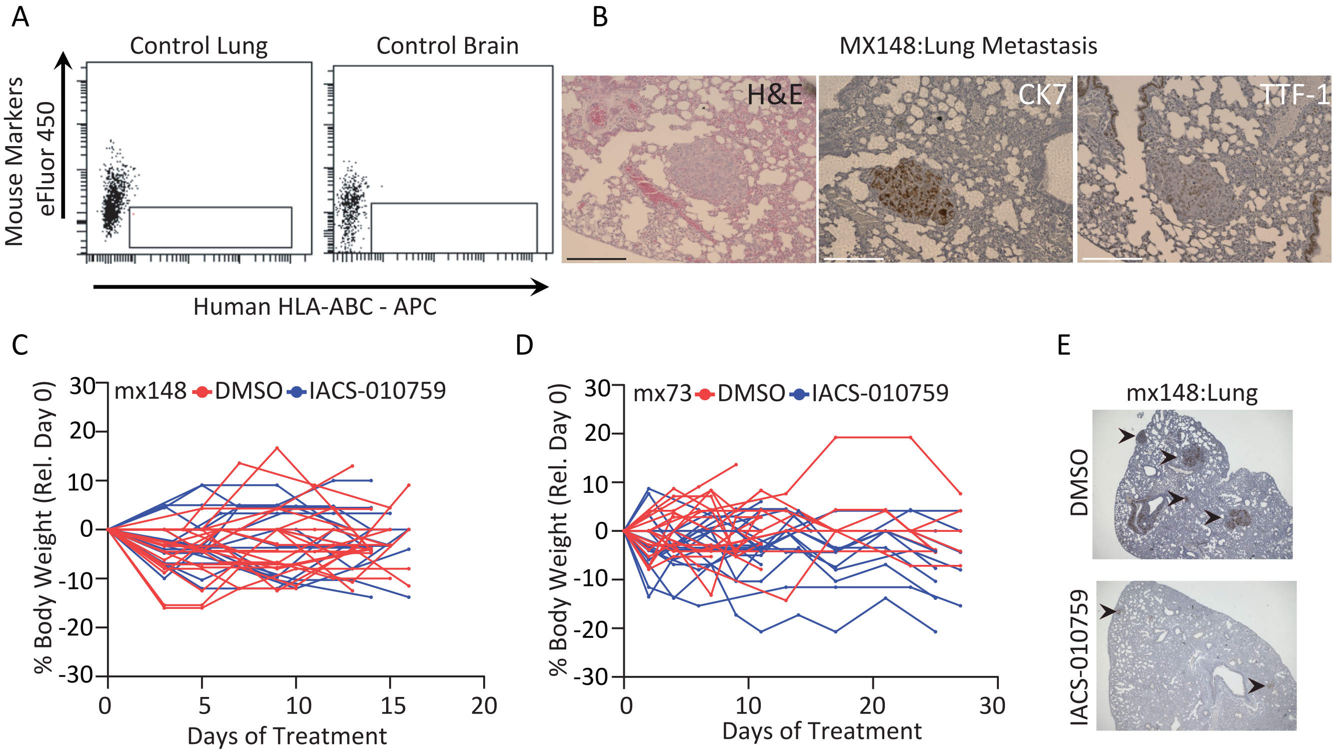

Supplementary Figure 6 (related to Figures 5 and 6): Treatment with IACS-010759 reduces distant metastasis. A) Flow cytometry plots from control (non-tumor bearing) NSG mice. B) Confirmation of NSCLC lung metastases from MX148 included H&E stain (scale bar 75µm), and IHC for CK7 and TTF-1 (scale bar is 150µm). C-D) Weights of individual DMSO- or IACS-010759-treated mice. Each mouse was weighed every 2-3 days and the weight was expressed as a percentage of the weight on day 0. E) Immunohistochemistry analysis of Ki67 in lung metastases from DMSO or IACS-010759 treated mice bearing mx148. Metastatic lesions are indicated by arrows.
